# Association between iron deficiency anemia and the risk of new-onset tuberculosis infection: a matched cohort analysis

**DOI:** 10.3389/fnut.2026.1727992

**Published:** 2026-01-20

**Authors:** I-Wen Chen, Li-Chen Chang, Ying-Jen Chang, Yi-Chen Lai, Kuo-Chuan Hung

**Affiliations:** 1 Department of Anesthesiology, Chi Mei Medical Center, Liouying, Tainan, Taiwan; 2 Department of Anesthesiology, E-Da Hospital, I-Shou University, Kaohsiung, Taiwan; 3 Department of Anesthesiology, Chi Mei Medical Center, Tainan, Taiwan

**Keywords:** extrapulmonary tuberculosis, iron deficiency anemia, risk factor, thrombocytosis, tuberculosis

## Abstract

**Background:**

Tuberculosis remains a major global health challenge, with emerging evidence suggesting that micronutrient deficiencies may contribute to susceptibility to infection through impaired immune function. Iron deficiency anemia (IDA) is the most common nutritional deficiency worldwide; however, its association with tuberculosis risk remains inadequately characterized across diverse populations. We examined the relationship between IDA and incident tuberculosis in a large multinational cohort.

**Methods:**

This retrospective matched cohort study used data from the TriNetX Research Network spanning 140 healthcare organizations across multiple countries between 2010 and 2020. We identified 177,846 adult patients with IDA and 309,662 controls with dermatitis. After one-to-one propensity score matching, each cohort comprised 160,928 patients. Primary outcomes included incident tuberculosis during 5-year and extended follow-up periods (5–10 years). We incorporated pneumonia and reactive thrombocytosis as positive control outcomes and performed subgroup analyses stratified by sex and age.

**Results:**

IDA was associated with increased tuberculosis risk during 5-year follow-up (hazard ratio [HR] 1.48, 95% confidence interval 1.10–2.00, *p* = 0.010), with attenuation during extended follow-up. The positive control outcomes demonstrated the expected robust associations with IDA, supporting internal validity. Subgroup analyses revealed numerically stronger associations in male patients (HR 2.06 versus 1.27 in females) and younger adults aged 18–50 years (HR 2.42 versus 1.47 in older individuals), although the interaction terms did not reach statistical significance (*p* = 0.193 and *p* = 0.268, respectively), suggesting no significant effect modification. Multivariate analysis revealed differential associations by tuberculosis subtype, with extrapulmonary tuberculosis demonstrating a more pronounced relationship (HR 3.01) than pulmonary disease (HR 1.71).

**Conclusion:**

IDA demonstrated a significant association with increased tuberculosis risk in this multinational cohort, particularly during early follow-up and for extrapulmonary manifestations. These findings suggest that IDA may represent an underrecognized modifiable risk factor for tuberculosis. Future research is warranted to validate these findings, with particular attention to the potential modifying effect of iron supplementation on tuberculosis risk.

## 1 Introduction

Tuberculosis continues to pose a major global health challenge, with an estimated 10.8 million new cases and 1.25 million deaths reported in 2023 (1–3). Despite substantial investment in tuberculosis control programs, tuberculosis control efforts have not achieved meaningful progress, with incidence reductions far below international goals. Pan et al. (4) reported that the global annual decline in tuberculosis incidence between 2015 and 2017 was limited to −1.1%, substantially below the projected 50% reduction target set for 2025. Similarly, MacNeil et al. (5) observed only a modest 2% decrease in global tuberculosis cases by 2018. According to the WHO Global Tuberculosis Report 2024, global TB incidence has decreased by only 8.3% and TB mortality by 23% since 2015, far below the End TB Strategy milestones of 50 and 75% reductions by 2025, indicating that TB has likely reverted to the world’s leading cause of death from a single infectious agent and remains a substantial unmet global health challenge (6). Well-established risk factors for tuberculosis include immunosuppression, HIV infection, diabetes mellitus, malnutrition, and chronic kidney disease (7–12). However, emerging evidence indicates that micronutrient deficiencies, particularly iron deficiency, may be under-recognized contributors to increased infection susceptibility through their adverse effects on cell-mediated immunity and macrophage function (13–16).

Iron deficiency anemia (IDA) represents the most common nutritional deficiency globally, with anemia affecting approximately one-third of the world’s population and accounting for roughly half of all anemia cases (17–19). While a landmark population-based study (20) demonstrated that IDA was associated with a nearly two-fold increased risk of tuberculosis in an Asian population, this cohort was derived from a single racially homogeneous Asian population. Accordingly, the results (20) may not be generalizable to other racial groups or geographic regions. Furthermore, the study (20) did not distinguish between pulmonary and extrapulmonary tuberculosis presentations, nor did it include validation measures, such as positive control outcomes, to ensure internal consistency of findings. Given that tuberculosis remains a global health priority affecting diverse populations worldwide, there is an urgent need to validate these associations in a large, multinational, ethnically diverse cohort with comprehensive subtype analysis. To address these knowledge gaps, we used a multinational database spanning multiple healthcare organizations, examining anatomic-specific tuberculosis presentations, and incorporating positive control outcomes to strengthen the validity of observed associations.

## 2 Methods

### 2.1 Study design and data source

This retrospective matched cohort study utilized data from the TriNetX Research Network, a federated health research platform that aggregates electronic health records from more than 140 healthcare organizations across the United States and other countries, including Australia, Belgium, Brazil, Bulgaria, Estonia, France, Georgia, Germany, Ghana, Israel, Italy, Japan, Lithuania, Malaysia, Poland, Singapore, Spain, Taiwan, the United Arab Emirates, and the United Kingdom. The network provides access to real-time clinical data encompassing patient demographics, diagnoses, procedures, laboratory results, and medication records. The TriNetX Research Network has been extensively used in numerous large-scale observational studies across diverse medical specialties, supporting the reliability and validity of its data for clinical research (21–24). All patient information underwent complete de-identification before inclusion in the database, ensuring compliance with privacy regulations and preventing the identification of individual patients. The study was approved by the Institutional Review Board of Chi Mei Medical Center (IRB No. 11403-E01), which granted a waiver of informed consent given the retrospective design and use of de-identified data.

### 2.2 Study population

The study enrolled adult patients aged 18 years or older who were documented in the TriNetX Research Network between January 1, 2010, and December 31, 2020. The exposed cohort consisted of individuals diagnosed with IDA (ICD-10 code D50) who received at least one additional IDA diagnosis within 2 years of the initial diagnosis to enhance diagnostic accuracy. The comparison cohort comprised patients whose first recorded diagnosis during the study period was unspecified dermatitis (ICD-10 code L30) with no documented history of IDA. Dermatitis was selected as the reference condition because it is a common, generally benign diagnosis that provides a clear index date, has no known biological link to iron metabolism or tuberculosis, and offers a neutral comparator population for cohort matching. To maintain mutual exclusivity between cohorts, patients with IDA who were diagnosed with dermatitis were excluded. The index date was defined as the date of the first diagnosis of either IDA or dermatitis.

To ensure the identification of new-onset tuberculosis cases, we applied several exclusion criteria. Patients were excluded if they had any tuberculosis diagnosis (ICD-10 codes A15-A19), latent tuberculosis infection (ICD-10 code Z22.7), documented contact with or suspected exposure to tuberculosis (ICD-10 code Z20.1), or use of anti-tuberculosis medications (isoniazid, rifampin, pyrazinamide, or ethambutol) before the index date. Additional exclusions included prior use of glucocorticoids, immunosuppressants, or antineoplastic agents; history of organ transplantation (ICD-10 code Z94); HIV infection (ICD-10 code B20); and other forms of anemia (ICD-10 codes D60-D64). To minimize reverse causation and detection bias, we excluded patients diagnosed with tuberculosis, latent tuberculosis, or tuberculosis exposure within 3 months following the index date.

### 2.3 Propensity score matching

We employed one-to-one propensity score matching using a greedy nearest-neighbor algorithm to balance the baseline characteristics between cohorts. The propensity score model incorporated demographic variables, including age at index, sex, race, and body mass index. Clinical covariates encompassed numerous comorbidities such as essential hypertension, lipoprotein metabolism disorders, neoplasms, diabetes mellitus, overweight and obesity, mood disorders, and ischemic heart disease. Mood disorders were included because they are frequently associated with nutritional deficiencies, altered health-seeking behavior, and impaired immune function, and have been linked to increased susceptibility to infectious diseases (25), thereby representing a potential confounder in the association between IDA and tuberculosis risk. Laboratory parameters included serum albumin, estimated glomerular filtration rate calculated by the CKD-EPI formula, and hemoglobin A1c. Medication use for diabetes management (biguanides, insulin analogs, GLP-1 analogs, and SGLT2 inhibitors) was also included. We assessed covariate balance using standardized mean differences, with values below 0.1 indicating adequate balance between matched cohorts.

### 2.4 Study outcomes

The primary outcome was incident tuberculosis infection occurring within 5 years after the index date. Secondary outcomes included tuberculosis incidence during the 5-to-10-year post-index period to examine temporal relationships. To validate the internal consistency, we incorporated pneumonia and reactive thrombocytosis as positive control outcomes, given their established associations with IDA in the previous literature. For exploratory analyses, we performed separate multivariate assessments examining IDA as a risk factor for pulmonary versus extrapulmonary tuberculosis, adjusting for sex, age, diabetes mellitus, chronic kidney disease, malnutrition, obesity, nicotine dependence, chronic obstructive pulmonary disease, hypertension, liver disease, and vitamin D deficiency.

### 2.5 Statistical analysis

Baseline characteristics are presented as means with standard deviations for continuous variables and frequencies with percentages for categorical variables. We evaluated the covariate balance between matched cohorts using standardized mean differences and visually confirmed balance by examining propensity score distributions before and after matching. The cumulative incidence of new-onset tuberculosis was estimated using the Kaplan–Meier method, with between-group comparisons performed using log-rank tests. Hazard ratios (HR) and corresponding 95% confidence intervals (CI) were calculated using Cox proportional hazards regression models. The proportional hazards assumption was verified through examination of Schoenfeld residuals. Subgroup analyses stratified by sex and age category (18–50 years versus over 50 years) were conducted to explore potential effect modification. Although the TriNetX cohort included patients from diverse racial backgrounds (e.g., White or Black or African American), subgroup analyses stratified by race were not performed because several racial strata had limited tuberculosis event counts, precluding stable estimation and adequate statistical power for interaction testing. Therefore, race was adjusted for as a covariate within the propensity score model rather than used as a stratification variable for subgroup analyses. Statistical significance was defined as a two-sided *p*-value less than 0.05. All analyses were performed within the TriNetX analytics platform using integrated statistical tools.

## 3 Result

### 3.1 Patient selection and baseline characteristics

**Figure 1:**
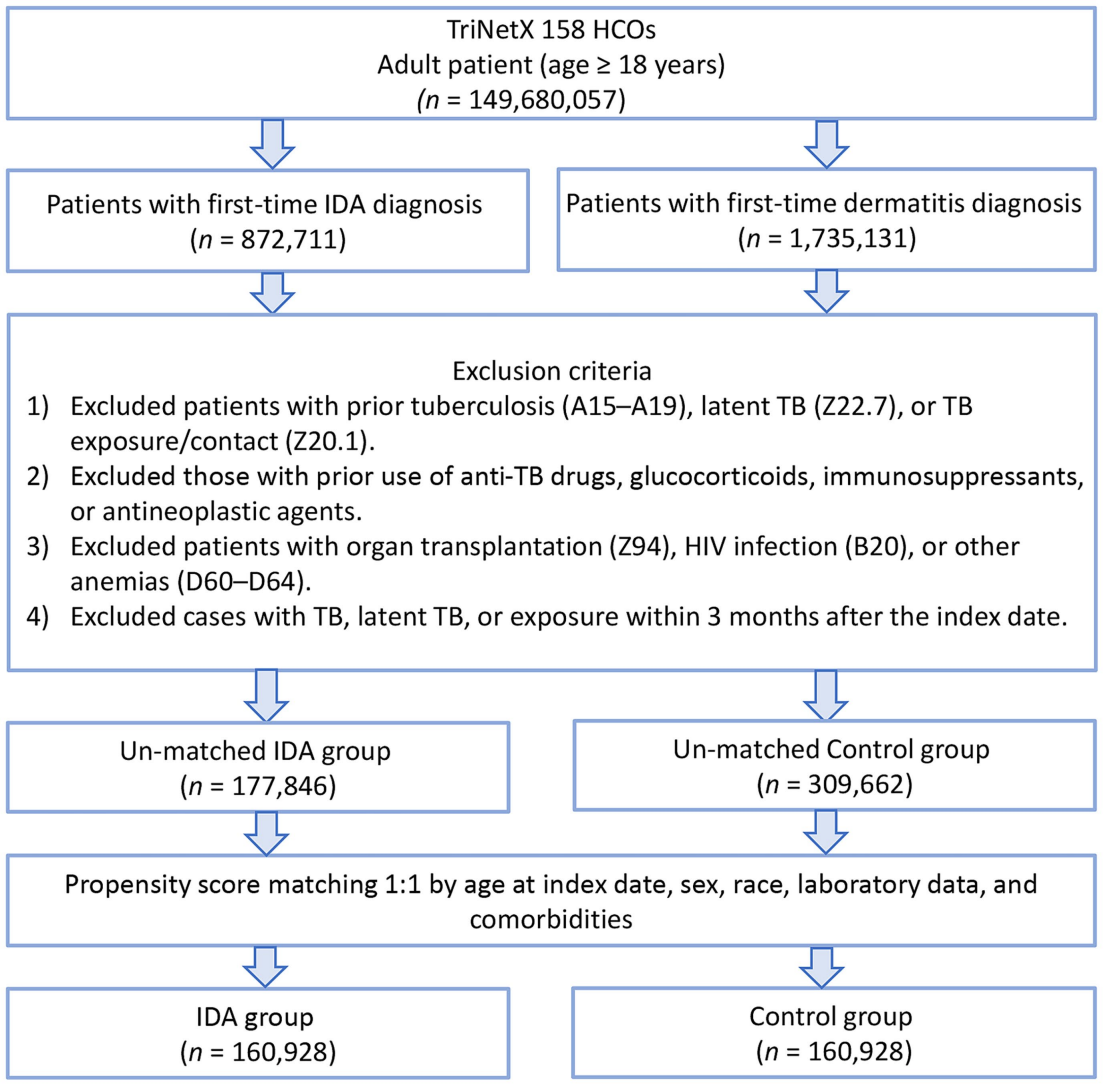
Patient selection flowchart from the TriNetX database. IDA, Iron deficiency anemia HCOs, Healthcare organizations; TB, Tuberculosis.

The initial cohort from the TriNetX Research Network included 177,846 patients with IDA and 309,662 patients with dermatitis as the control group (Figure 1). After performing one-to-one propensity score matching using a greedy nearest-neighbor algorithm, the final matched cohort comprised 160,928 patients. Before matching, substantial imbalances were evident between the groups across multiple domains (Table 1). The IDA cohort demonstrated notably higher mean age (52.7 versus 41.9 years) and a lower proportion of male patients (27.6% versus 44.5%). The matched cohort was predominantly composed of White patients (approximately 46–48%), with substantially smaller proportions of Black or African American (approximately 12–15%) and Asian (approximately 5%) individuals, which further limited the feasibility of race-specific subgroup analyses. Marked differences were also observed in the prevalence of cardiovascular and metabolic comorbidities, with the IDA group showing higher rates of essential hypertension (17.8% vs. 9.8%), diabetes mellitus (9.7% vs. 4.5%), ischemic heart disease (6.3% vs. 2.1%), and chronic kidney disease (3.8% vs. 1.1%).

**Table 1:** Baseline characteristics of patients before and after propensity score matching.

Variables	Before matching			After matching		
	IDA group (*n* = 177,846)	Control group (*n* = 309,662)	SMD†	IDA group (*n* = 160,928)	Control group (*n* = 160,928)	SMD†
Patient characteristics						
Age at index (years)	52.7 ± 21.8	41.9 ± 22.2	0.492	50.8 ± 21.4	51.9 ± 21.1	0.048
Male	49,146 (27.6)	137,703 (44.5)	0.356	46,780 (29.1)	43,668 (27.1)	0.043
BMI > 30 kg/m2	28.6 ± 8.0	27.7 ± 7.3	0.158	28.7 ± 7.9	28.8 ± 7.7	0.019
White	81,577 (45.9)	151,866 (49.0)	0.064	74,889 (46.5)	77,707 (48.3)	0.035
Black or African American	29,517 (16.6)	30,011 (9.7)	0.205	23,579 (14.7)	19,300 (12.0)	0.078
Asian	8,695 (4.9)	14,922 (4.8)	0.003	8,135 (5.1)	8,319 (5.2)	0.005
Factors influencing health status and contact with health services	62,717 (35.3)	95,264 (30.8)	0.096	54,810 (34.1)	57,184 (35.5)	0.031
Comorbidities (ICD-10 code)						
Essential (primary) hypertension (I10)	31,733 (17.8)	30,473 (9.8)	0.233	24,535 (15.2)	24,337 (15.1)	0.003
Dyslipidemia (E78)	20,620 (11.6)	27,652 (8.9)	0.088	17,357 (10.8)	18,567 (11.5)	0.024
Neoplasms (C00-D49)	16,004 (9.0)	26,565 (8.6)	0.015	14,515 (9.0)	16,013 (10.0)	0.032
Diabetes mellitus (E08-E13)	17,312 (9.7)	13,834 (4.5)	0.206	12,637 (7.9)	11,795 (7.3)	0.020
Overweight and obesity (E66)	10,383 (5.8)	14,012 (4.5)	0.059	8,913 (5.5)	9,395 (5.8)	0.013
Mood [affective] disorders (F30-F39)	9,896 (5.6)	14,077 (4.5)	0.046	8,622 (5.4)	9,453 (5.9)	0.022
Ischemic heart diseases (I20-I25)	11,127 (6.3)	6,582 (2.1)	0.207	7,301 (4.5)	6,172 (3.8)	0.035
Hypothyroidism (E03)	8,543 (4.8)	8,710 (2.8)	0.104	7,015 (4.4)	7,216 (4.5)	0.006
Vitamin D deficiency (E55)	7,827 (4.4)	8,798 (2.8)	0.084	6,595 (4.1)	6,925 (4.3)	0.010
Sleep disorders (G47)	6,732 (3.8)	10,913 (3.5)	0.014	5,965 (3.7)	6,653 (4.1)	0.022
Nicotine dependence (F17)	5,529 (3.1)	6,548 (2.1)	0.062	4,499 (2.8)	4,790 (3.0)	0.011
Chronic kidney disease (CKD) (N18)	6,788 (3.8)	3,370 (1.1)	0.177	4,140 (2.6)	3,221 (2.0)	0.038
Diseases of liver (K70-K77)	4,017 (2.3)	3,337 (1.1)	0.092	3,024 (1.9)	2,853 (1.8)	0.008
COPD (J44)	3,891 (2.2)	2,579 (0.8)	0.111	2,706 (1.7)	2,375 (1.5)	0.017
Obstructive sleep apnea (G47.33)	2,821 (1.6)	3,788 (1.2)	0.031	2,389 (1.5)	2,494 (1.6)	0.005
Cerebral infarction (I63)	2,320 (1.3)	1707 (0.6)	0.079	1,651 (1.0)	1,518 (0.9)	0.008
Systemic connective tissue disorders (M30-M36)	1,095 (0.6)	1,252 (0.4)	0.030	909 (0.6)	966 (0.6)	0.005
Malnutrition (E40-E46)	808 (0.5)	383 (0.1)	0.062	492 (0.3)	367 (0.2)	0.015
Lung diseases due to external agents (J60-J70)	435 (0.2)	399 (0.1)	0.027	326 (0.2)	318 (0.2)	0.001
Interstitial pulmonary diseases (J84)	408 (0.2)	330 (0.1)	0.030	307 (0.2)	283 (0.2)	0.003
Laboratory data						
Hemoglobin A1c ≥ 7%	5,415 (3.0)	5,187 (1.7)	0.090	4,173 (2.6)	4,079 (2.5)	0.004
Albumin g/dL (≥3.5 g/dL)	36,602 (20.6)	42,150 (13.6)	0.186	30,262 (18.8)	31,487 (19.6)	0.019
eGFR>60 mL/min/1.73 m^2^	41,237 (23.2)	42,505 (13.7)	0.246	33,246 (20.7)	33,608 (20.9)	0.006
Medication						
Biguanides	5,610 (3.2)	4,734 (1.5)	0.108	4,167 (2.6)	4,006 (2.5)	0.006
Insulins and analogues	5,196 (2.9)	3,274 (1.1)	0.134	3,427 (2.1)	2,908 (1.8)	0.023
GLP-1 analogues	515 (0.3)	511 (0.2)	0.026	413 (0.3)	421 (0.3)	0.001
SGLT2 inhibitors	379 (0.2)	479 (0.2)	0.014	332 (0.2)	347 (0.2)	0.002

IDA, Iron deficiency anemia; BMI, Body mass index; SMD, Standardized mean differences; †SMD values <0.1 indicate adequate balance between groups; eGFR, estimated Glomerular Filtration Rate; GLP-1, Glucagon-like peptide-1; SGLT2, Sodium-glucose co-transporter 2; COPD, chronic obstructive pulmonary disease.

**Figure 2:**
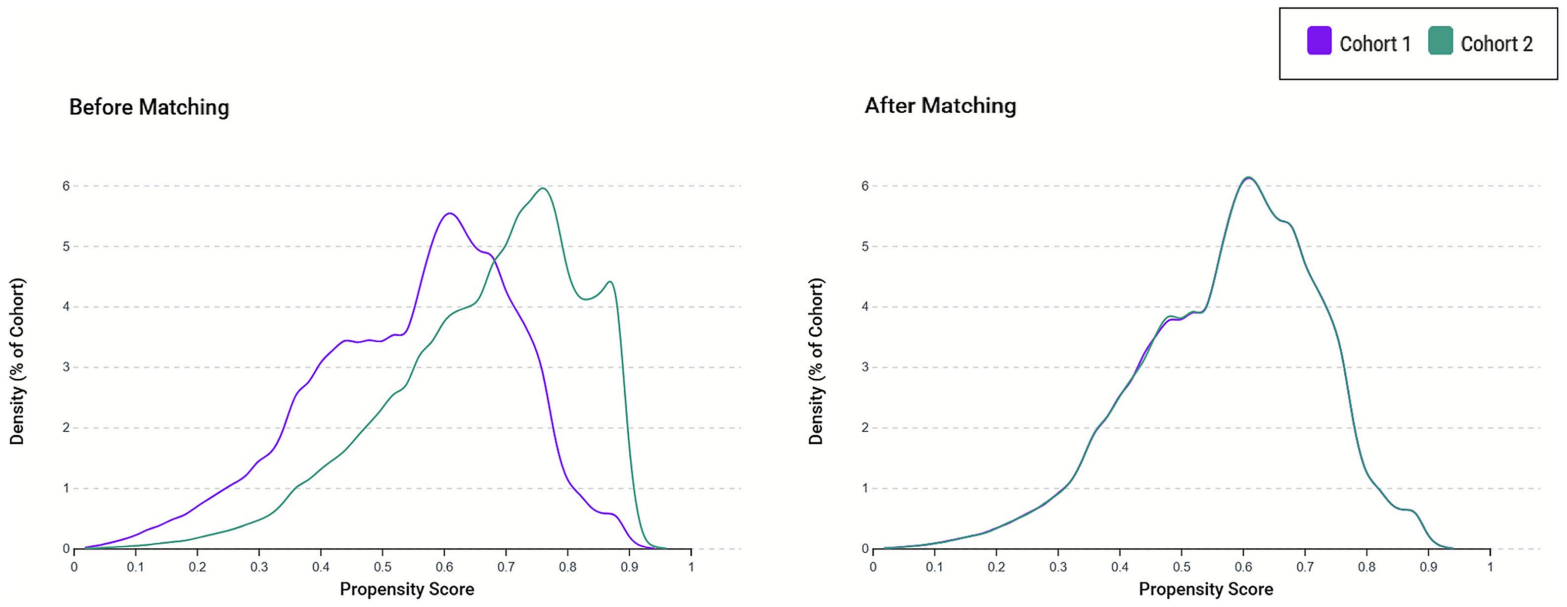
Propensity score density distributions before and after matching. The left panel displays the propensity score distributions for the iron deficiency anemia (IDA) cohort (Cohort 1, purple) and control cohorts (Cohort 2, teal) prior to matching, demonstrating substantial baseline differences in the probability of exposure. The right panel shows overlapping propensity score distributions after 1:1 greedy nearest-neighbor matching, indicating a successful balance between cohorts.

Following propensity score matching, an excellent covariate balance was achieved across all measured baseline characteristics, effectively eliminating the substantial differences observed in the unmatched cohorts (Table 1). The matched cohorts demonstrated comparable demographic profiles, with similar mean ages (50.8 versus 51.9 years) and sex distribution (29.1% versus 27.1% male). Racial composition, body mass index distributions, prevalence of all assessed comorbidities, laboratory parameters, and medication usage patterns were well balanced between the groups. Visual inspection of the propensity score density distributions (Figure 2) further confirmed the achievement of overlap and balance between the two groups following the matching procedure.

### 3.2 Primary outcome: tuberculosis infection

During the 5-year follow-up, a higher occurrence of tuberculosis was observed among patients with IDA (108 vs. 70 cases), corresponding to incidence rates of 22 and 14 per 100,000 person-years, respectively. IDA was associated with an increased likelihood of developing tuberculosis (HR 1.48, 95% CI 1.10–2.00, *p* = 0.010). However, this association appeared to weaken over time, as the 5–10-year follow-up showed no significant difference between the two cohorts (21 vs. 19 events; HR 1.17, 95% CI 0.63–2.17, *p* = 0.627). These results suggest a time-dependent association, with elevated tuberculosis risk primarily evident in the early years after IDA diagnosis.

Both positive control outcomes demonstrated robust associations with IDA, thus supporting the internal validity of the study findings. The incidence of pneumonia was substantially higher in the IDA cohort during both the 5-year follow-up period (4.6% versus 2.4%; HR, 1.87; 95% CI, 1.79–1.94; *p* < 0.001) and the 5-to-10-year period (0.96% versus 0.64%; HR, 1.60; 95% CI, 1.48–1.73; *p* < 0.001). Similarly, reactive thrombocytosis showed a marked association with IDA at 5 years (0.31% versus 0.09%; HR, 3.68; 95% CI, 3.04–4.46; *p* < 0.001) and remained significant in the extended follow-up (0.12% versus 0.06%; HR, 2.06; 95% CI, 1.61–2.63; *p* < 0.001; Table 2).

**Table 2:** Five-year and extended follow-up outcomes in matched cohorts with and without iron deficiency anemia.

Outcomes	IDAgroup (*n* = 160,928)	Control group (*n* = 160,928)	HR (95% CI)	*p*-value
5-year follow-up				
TB infection, Events (IR/100,000 PY)	108 (22)	70 (14)	1.48 (1.10–2.00)	0.010
Pneumonia, event (%)	7,346 (4.6%)	3,831 (2.4%)	1.87 (1.79–1.94)	<0.001
Reactive thrombocytosis event (%)	495 (0.31%)	131 (0.09%)	3.68 (3.04–4.46)	<0.001
5–10 year follow-up				
TB infection, Events (IR/100,000 PY)	21 (3)	19 (3)	1.17 (0.63–2.17)	0.627
Pneumonia event (%)	1,545 (0.96%)	1,024 (0.64%)	1.60 (1.48–1.73)	<0.001
Reactive thrombocytosis event (%)	187 (0.12%)	96 (0.06%)	2.06 (1.61–2.63)	<0.001

TB, Tuberculosis; IDA, Iron deficiency anemia; HR, Hazard ratio; CI, Confidence interval; IR, Incidence rate; PY, Person-years.

### 3.3 Subgroup analyses

Sex-stratified analyses revealed potential effect modification, with male patients demonstrating a stronger association between IDA and tuberculosis risk during the 5-year follow-up period (HR, 2.06; 95% CI, 1.26–3.41; *p* = 0.003) compared with female patients (HR, 1.27; 95% CI, 0.86–1.88; *p* = 0.235), although the interaction term did not reach statistical significance (*p* for interaction = 0.193; Table 3). Age stratification demonstrated that younger adults aged 18 to 50 years exhibited a more pronounced association (HR, 2.42; 95% CI, 1.31–4.49; *p* = 0.004) relative to those older than 50 years (HR, 1.47; 95% CI, 1.02–2.12; *p* = 0.036), although again the interaction was not statistically significant (*p* for interaction = 0.268; Table 4). This finding indicated that the association between IDA and tuberculosis risk did not differ significantly across age groups, and that no statistically significant effect modification by age was observed.

**Table 3:** Sex-stratified analysis of tuberculosis and related outcomes in patients with iron deficiency anemia.

Outcomes	Female (*n* = 115,361)		Male (*n* = 42,082)		*p* for interaction
	HR (95% CI)	*p*-values	HR (95% CI)	*p*-values	
5-year follow-up					
New-onset TB infection	1.27 (0.86–1.88)	0.235	2.06 (1.26–3.41)	0.003	0.193
Pneumonia	1.70 (1.62–1.80)	<0.001	2.00 (1.88–2.12)	<0.001	<0.001
Reactive thrombocytosis	3.16 (2.57–3.88)	<0.001	4.10 (2.62–6.41)	<0.001	0.358
5-10-year follow-up					
New-onset TB infection	1.67 (0.79–3.54)	0.174	NA	NA	NA
Pneumonia	1.48 (1.34–1.64)	<0.001	1.79 (1.58–2.04)	<0.001	0.027
Reactive thrombocytosis	2.06 (1.57–2.71)	<0.001	2.19 (1.18–4.07)	0.011	0.870

TB, Tuberculosis; HR, Hazard ratio; CI, Confidence interval; *p* for interaction indicates statistical significance of effect modification by sex. NA, not available; based on the outcome terms, the patient count was too small for detailed results to be displayed.

**Table 4:** Age-stratified analysis of tuberculosis and related outcomes in patients with iron deficiency anemia.

5-year follow-up	18–50 yr. (*n* = 55,302)		>51 (*n* = 98,313)		P for interaction
	HR (95% CI)	*p*-values	HR (95% CI)	*p*-values	
5-year follow-up					
New-onset TB infection	2.42 (1.31–4.49)	0.004	1.47 (1.02–2.12)	0.036	0.268
Pneumonia	1.48 (1.32–1.67)	<0.001	1.98 (1.90–2.07)	<0.001	<0.001
Reactive thrombocytosis	4.11 (2.98–5.69)	<0.001	3.17 (2.50–4.03)	<0.001	0.262
5-10-year follow-up					
New-onset TB infection	NA	NA	1.80 (0.86–3.77)	0.117	NA
Pneumonia	1.45 (1.15–1.81)	0.001	1.63 (1.49–1.77)	<0.001	0.325
Reactive thrombocytosis	2.66 (1.72–4.13)	<0.001	1.71 (1.24–2.36)	0.001	0.161

TB, Tuberculosis; HR, Hazard ratio; CI, Confidence interval; *p* for interaction indicates statistical significance of effect modification by age. NA, not available; based on the outcome terms, the patient count was too small for detailed results to be displayed.

### 3.4 Multivariate analysis of tuberculosis subtypes

Adjusted multivariate models examining tuberculosis subtypes revealed differential associations between IDA and disease presentation (Table 5). IDA was associated with an increased risk of both pulmonary tuberculosis (HR, 1.71; 95% CI, 1.30–2.24; *p* < 0.001) and extrapulmonary tuberculosis (HR, 3.01; 95% CI, 1.73–5.22; *p* < 0.001), with the association appearing stronger for extrapulmonary manifestations. Male sex independently predicted both pulmonary (HR, 1.62; 95% CI, 1.24–2.10; *p* < 0.001) and extrapulmonary tuberculosis (HR, 1.73; 95% CI, 1.03–2.92; *p* = 0.039). Advancing age was associated with pulmonary tuberculosis risk (HR, 1.01; 95% CI, 1.01–1.02; *p* = 0.001) but not extrapulmonary disease.

**Table 5:** Multivariable analysis of risk factors for pulmonary and extrapulmonary tuberculosis.

Variable	Pulmonary TB		Extra-pulmonary TB	
	HR (95% CI)	*p*-value	HR (95% CI)	*p*-value
IDA or control groups	1.71 (1.30, 2.24)	<0.001	3.01 (1.73, 5.22)	<0.001
Male	1.62 (1.24, 2.10)	<0.001	1.73 (1.03, 2.92)	0.039
Age at Index	1.01 (1.01, 1.02)	0.001	1.01 (0.99, 1.02)	0.438
Diabetes mellitus	0.82 (0.49, 1.38)	0.451	0.68 (0.19, 2.44)	0.558
Chronic kidney disease	1.85 (0.99, 3.47)	0.055	2.93 (0.83, 10.42)	0.096
Malnutrition	1.56 (0.22, 11.26)	0.657	NA	NA
Overweight and obesity	0.49 (0.23, 1.06)	0.069	NA	NA
Nicotine dependence	1.13 (0.55, 2.34)	0.736	1.49 (0.36, 6.20)	0.584
Chronic obstructive pulmonary disease	1.09 (0.44, 2.73)	0.855	NA	NA
Essential (primary) hypertension	0.99 (0.67, 1.46)	0.957	0.58 (0.22, 1.50)	0.256
Diseases of liver	1.13 (0.46, 2.76)	0.793	1.04 (0.14, 7.60)	0.971
Vitamin D deficiency	0.91 (0.46, 1.78)	0.776	NA	NA

TB, Tuberculosis; HR, Hazard ratio; CI, Confidence interval; NA, Not available, the HR could not be reliably estimated due to sparse data or quasi-complete separation.

## 4 Discussion

This large multinational matched cohort study demonstrates that IDA is associated with an increased risk of subsequent tuberculosis, supporting the hypothesis that IDA may contribute to vulnerability to mycobacterial infection. Rather than reflecting a coincidental association, our findings indicate that IDA may represent a clinically relevant marker of susceptibility during the early disease-prone period following diagnosis, when immune dysfunction is likely most pronounced. The consistent associations observed across positive control outcomes further support the validity of this relationship. Importantly, the stronger association with extrapulmonary tuberculosis suggests that IDA may be particularly linked to impaired systemic immune containment, potentially facilitating hematogenous dissemination beyond pulmonary involvement. Collectively, these findings extend prior single-population observations to a diverse international cohort and reinforce the emerging concept that IDA plays a meaningful role in tuberculosis risk.

The observed association between IDA and tuberculosis in our study aligns with emerging evidence regarding the role of iron homeostasis in immune function and susceptibility (13–16). Iron has dual biological functions, acting as an essential cofactor for both host immune responses and mycobacterial metabolism (15). The impairment of cell-mediated immunity associated with iron deficiency may contribute to increased vulnerability to tuberculosis infection, as effective control of mycobacterial infection depends heavily on T-cell-mediated responses and macrophage activation (26–28). Our findings demonstrated that patients with IDA were associated with a nearly 50% increased risk. The inclusion of positive control outcomes, pneumonia, and reactive thrombocytosis, both of which showed expected strong associations with IDA, provided methodological validation of our analytical approach and strengthened confidence in the tuberculosis findings. These positive controls confirmed that our study design successfully captured the known associations between IDA and related health outcomes.

Our investigation advances beyond prior research through several methodological improvements that directly address earlier limitations. The landmark population-based study (20), conducted within an Asian population using a single-country administrative database, provided important initial evidence but was constrained by its ethnically homogeneous cohort and limited generalizability. In contrast, our study leveraged the TriNetX Research Network, which integrates electronic health records from over 140 healthcare organizations spanning multiple countries. This multinational and ethnically diverse cohort—including substantial proportions of White, Black, or African American, and Asian participants—enabled a broader and more representative assessment of the association between IDA and tuberculosis risk across diverse demographic groups. Furthermore, we incorporated positive control outcomes as a validation strategy, a methodological approach absent from previous studies. The distinction between pulmonary and extrapulmonary tuberculosis in multivariate analyses represents another novel contribution, as a prior study (20) did not differentiate between anatomic presentations of tuberculosis disease. Our substantially larger matched cohort of 160,928 patients per group, compared with previous sample sizes (20), provided enhanced statistical power for subgroup analyses and exploration of effect modification.

The temporal relationship observed in our study demonstrated consistency with previous findings (20), while providing additional granularity regarding the duration of association. An earlier population study (20) reported that associations were strongest within the first 2 years following IDA diagnosis. Our findings corroborated this temporal pattern, as the elevated tuberculosis risk in our cohort was primarily concentrated in the initial 5 years after IDA diagnosis, with attenuation during the five-to-ten-year follow-up period. Several biological mechanisms may explain this time-dependent relationship. The acute immunological consequences of iron deficiency, including impaired lymphocyte proliferation and reduced natural killer cell activity (29, 30), may exert their strongest effects during active iron depletion. Additionally, many patients are likely to receive iron supplementation following diagnosis, potentially restoring immune function over time and diminishing the subsequent tuberculosis risk. The early concentration of tuberculosis cases may also reflect detection bias, as patients with newly diagnosed IDA undergo more frequent medical evaluation, potentially facilitating earlier tuberculosis diagnosis. Nevertheless, by incorporating a three-month exclusion period after the index date to minimize detection bias, our study design strengthens the inference that the early association observed represents a true elevation in tuberculosis susceptibility rather than a spurious finding due to differential diagnosis timing.

Examining sex-specific patterns, male patients demonstrated a stronger association between IDA and tuberculosis than female patients, although the interaction term did not achieve statistical significance. This finding paralleled observations from the prior study (20), which reported adjusted hazard ratios of 2.02 for men and 1.96 for women. While an earlier study demonstrated nearly equivalent estimates between sexes, our analysis revealed a more substantial point estimate difference, although overlapping confidence intervals suggest that these differences may reflect sampling variation rather than true population heterogeneity. Age stratification revealed that younger adults aged 18–50 years exhibited a more pronounced association, with a hazard ratio of 2.42 compared with 1.47 for those older than 50 years. The mechanisms underlying the observed age-related differences in the association between IDA and tuberculosis risk remain unclear at present. Further mechanistic and epidemiological studies are warranted to clarify the biological and clinical factors that may contribute to the differential susceptibility observed across age groups.

Multivariate models examining pulmonary versus extrapulmonary tuberculosis revealed differential associations that provide important insights into potential mechanisms. Extrapulmonary tuberculosis demonstrated a stronger association with IDA than with pulmonary disease. This distinction had not been previously examined in a population-based study (20) and represents a clinically relevant finding. Extrapulmonary tuberculosis typically reflects the hematogenous dissemination of mycobacteria and may indicate a more profound impairment of cell-mediated immunity. The stronger association observed with extrapulmonary manifestations suggests that IDA may particularly compromise systemic immune surveillance mechanisms that ordinarily prevent mycobacterial dissemination beyond the lungs.

The optimal exposure duration of IDA required to influence tuberculosis development remains undefined. In the present study, exposure was operationalized as the diagnosis of IDA at baseline, and tuberculosis incidence was evaluated longitudinally without imposing a minimum exposure time threshold. Consequently, our analysis reflects real-world post-diagnosis risk rather than establishing a causal latency interval. Future prospective studies with detailed exposure characterization are required to define the temporal relationship and identify any critical duration of IDA associated with tuberculosis susceptibility.

Several methodological limitations should be considered when interpreting these findings. First, the reliance on diagnostic codes from electronic health records introduces potential for misclassification, although our requirement for secondary IDA diagnoses aimed to enhance diagnostic accuracy. Despite rigorous propensity score matching, residual confounding remains possible because certain potentially influential variables, including socioeconomic status, comprehensive nutritional status, and detailed smoking history, were not available in the database. Second, the inability to quantify iron deficiency severity or distinguish between different etiologies of IDA limits our capacity to examine dose–response relationships or identify particularly high-risk subgroups. Third, detection bias, whereby patients with IDA undergo more frequent medical evaluations leading to increased tuberculosis diagnosis, represents a concern despite our three-month exclusion period. Fourth, given the observational nature of this study, the associations identified should be interpreted with caution and should not be construed as evidence of a causal relationship between IDA and tuberculosis. Accordingly, IDA should be considered a clinical marker of increased susceptibility rather than a proven causal determinant of tuberculosis risk. Fifth, although this study leveraged a large multinational database encompassing diverse healthcare systems, the TriNetX Research Network predominantly comprises healthcare organizations from high-income countries, particularly the United States. As a result, the generalizability of these findings to regions with a higher tuberculosis burden, limited healthcare access, or differing nutritional and infectious disease epidemiology may be constrained. Future studies conducted in tuberculosis-endemic, resource-limited settings are warranted to confirm the applicability of these findings across global populations. Finally, information regarding iron supplementation after the diagnosis of IDA was incomplete and inconsistently recorded within the TriNetX database across participating institutions. As a result, we were unable to reliably ascertain treatment status, duration of exposure, or post-diagnosis iron repletion trajectories. This limitation precluded adjustment for iron supplementation as a time-varying exposure and may have led to underestimation of the true association between IDA and tuberculosis risk.

## 5 Conclusion

This large-scale multinational cohort study demonstrated an association between IDA and increased tuberculosis risk, with particularly pronounced relationships observed during early follow-up among younger adults and for extrapulmonary disease manifestations. These findings support an association between IDA and subsequent tuberculosis risk and highlight IDA as a potential clinical marker of heightened susceptibility. Further prospective and interventional studies are required to clarify causal pathways and to determine whether correction of IDA modifies tuberculosis risk.

## Data Availability

The raw data supporting the conclusions of this article will be made available by the authors, without undue reservation.
